# Prevalence and correlates of anxiety disorders and depressive disorders among older adults with non-communicable diseases: results from China Mental Health Survey

**DOI:** 10.3389/fpsyt.2025.1626540

**Published:** 2025-08-08

**Authors:** Hongmin Ge, Zhaorui Liu, Yue Tong, Yueqin Huang, Xiaofei Hou, Minghui Li, Yongping Yan, Shuiyuan Xiao, Lingjiang Li, Tingting Zhang, Jie Yan, Yaqin Yu, Xiufeng Xu, Zhizhong Wang, Yifeng Xu, Tao Li, Xiangdong Xu, Limin Wang, Huifang Yin, Guangming Xu

**Affiliations:** ^1^ Tianjin Anding Hospital, Mental Health Center of Tianjin Medical University, Tianjin, China; ^2^ Peking University Sixth Hospital, Peking University Institute of Mental Health, NHC Key Laboratory of Mental Health (Peking University), National Clinical Research Center for Mental Disorders (Peking University Sixth Hospital), Beijing, China; ^3^ Department of Epidemiology, Ministry of Education Key Lab of Hazard Assessment and Control in Special Operational Environment, School of Public Health, Fourth Military Medical University, Xi’an, China; ^4^ Department of Social Medicine and Health Management, School of Public Health, Central South University, Changsha, Hunan, China; ^5^ Mental Health Institute, The Second Xiangya Hospital, Central-South University, Changsha, Hunan, China; ^6^ Institute of Social Science Survey, Peking University, Beijing, China; ^7^ Department of Epidemiology and Biostatistics, School of Public Health, Jilin University, Changchun, Jilin, China; ^8^ Department of Psychiatry, The First Affiliated Hospital of Kunming Medical University, Kunming, Yunnan, China; ^9^ Department of Epidemiology and Statistics, School of Public Health and Management, Ningxia Medical University, Yinchuan, Ningxia, China; ^10^ Shanghai Mental Health Center, School of Medicine, Shanghai Jiao Tong University, Shanghai, China; ^11^ Mental Health Center, West China Hospital, Sichuan University, Chengdu, Sichuan, China; ^12^ The Fourth People’s Hospital in Urumqi, Urumqi, China; ^13^ National Center for Chronic and Non-communicable Disease Control and Prevention, Chinese Center for Disease Control and Prevention, Beijing, China

**Keywords:** anxiety disorders, depressive disorders, non-communicable diseases, cross-sectional study, epidemiology

## Abstract

**Background:**

Anxiety disorders (ADs) and depressive disorders (DDs) are prevalent among older adults with non-communicable diseases (NCDs), yet nationally representative epidemiological data from China remain limited. This study, as a part of the 2013–2015 China Mental Health Survey (CMHS), aimed to assess the prevalence of ADs and DDs and identify associated factors among older adults with NCDs in China.

**Method:**

A total of 5,428 patients aged 60 years and above with reported NCDs were selected from the CMHS. The Composite International Diagnostic Interview (CIDI) was used to diagnose ADs, DDs, and alcohol use disorders (AUDs) in order to assess their disease status and the lifetime and 12-month prevalence. Data were weighted by age, sex, and residential distribution from the 2010 Chinese census to correct for selection probabilities and response rates, and were post-stratified for national representativeness. Univariate and multivariate logistic regression was used to identify factors associated with 12-month ADs and DDs.

**Result:**

The lifetime prevalence of ADs was 8.3% and the 12-month prevalence of ADs was 5.8%. The two most prevalent ADs were specific phobia (SP; lifetime prevalence of 4.2% and 12-month prevalence of 3.1%) and obsessive-compulsive disorder (OCD; lifetime prevalence of 2.8% and 12-month prevalence of 2.1%). The lifetime prevalence of DDs was 10.1%, while the 12-month prevalence was 5.4%. The two most common DDs were major depressive disorder (MDD; lifetime prevalence of 7.2% and 12-month prevalence of 4.4%) and dysthymia (lifetime prevalence of 2.8% and 12-month prevalence of 2.2%). ADs are most common in individuals with heart attacks (12%), chronic lung disease (11.2%), and other chronic pain (11%). DDs are most commonly linked to heart attacks (13.8%), other chronic pain (13.2%), and cancer (13.1%). Female gender, alcohol use disorder, and comorbid with three or more NCDs were found to be risk factors associated with ADs and DDs.

**Conclusion:**

The co- occurrence of mental disorders among older adults with NCDs has become a growing public health concern in China. Primary health care institutions and clinicians should simultaneously enhance the management of chronic NCDs and mental health care among older adults.

## 1 Introduction

As the aging population increases, their physical and mental health has emerged as a significant global public health concern. Older adults often suffer from non-communicable diseases (NCDs), which are usually chronic and progress gradually. Previous studies indicate that approximately 85% of older adults have at least one chronic illness, while 60% have two or more in the US (1). Similarly, in China, 81.1% of older adults are affected by chronic diseases (2). Furthermore, NCDs are the primary cause of death, disability and comorbidities among older adults (3, 4).

Individuals with NCDs face significant mental health challenges and have much higher rates of anxiety and depression than the general population. A cohort study in China found that individuals with NCDs show much higher rates of anxiety and depressive symptoms than those without NCDs (5). Anxiety and depression share risk factors and biological pathways with NCDs. When these mental health issues co-occur with NCDs, the impact on patient outcomes is greater than when each condition occurs independently. Firstly, anxiety and depression can hinder the recovery process of individuals with NCDs and ultimately affect their quality of life (6). In addition, anxiety and depression can increase the risk of developing other physical health issues in individuals with NCDs (7). Moreover, severe anxiety and depression significantly increase the risk of suicide and contribute to higher mortality rates among individuals with NCDs (8). Existing literature identifies several sociodemographic factors, life styles and the number of chronic diseases as significant correlates of anxiety and depression in NCD patients (9, 10). However, the evidence regarding these factors is inconsistent. Given the high prevalence of NCDs in China, it is vital to clarify the risk factors for anxiety and depression among older adults with NCDs.

While the link between NCDs and anxiety and depression has been studied among older adults in China, there are still evidence gaps. First, previous studies have predominantly been geographically limited to specific regions (9, 11) rather than being nationally representative. Second, existing researches mainly have focused on depressive and anxiety symptoms (11, 12) rather than DDs and ADs. Although several studies have investigated DDs in Chinese older adults with NCDs (13), existing research has primarily focused on DDs in those with a specific NCD. For instance, a study examining DDs in Chinese older adults with essential hypertension reported a 1-month prevalence of 25.7% (14). There remains a need for a comprehensive overview of ADs and DDs across different NCDs.

To address these limitations, the nationally representative China Mental Health Survey (CMHS) were conducted to provide comprehensive data on the prevalence and correlates of ADs and DDs among community-dwelling older adults with NCDs in China. Based on CMHS, the objectives of this study are: (1) to assess the prevalence of ADs and DDs among a representative sample of older adults with different types of NCDs in China; (2) to identify factors related to these disorders.

## 2 Methods

### 2.1 Study design and population

This study utilized data from the CMHS, a nationally representative household epidemiological survey conducted from July 2013 to March 2015. The survey aimed to investigate the prevalence of mental disorders and dementia, gather data on service use among individuals with mental disorders in China, and analyze the social and psychological risk factors associated with mental disorders and mental health services (15). The design and procedures of the CMHS, which include sampling methods and diagnostic assessments, have been described (15–17). In brief, the CMHS was a cross-sectional study employing proportional stratified random sampling to select 40,964 participants from 31 provinces in China. A total of 28,140 eligible participants completed the Composite International Diagnostic Interview-3.0 (CIDI), including 7,454 individuals aged 60 and above. Among the individuals aged 60 years and above 5,428 were reported to have chronic physical diseases.

### 2.2 Procedures

The CMHS was conducted in two stages. Stage I focused on trained non-professional interviewers conducting face-to-face computer-assisted personal interviews (CAPI) with all respondents using CIDI to diagnose mental disorders including mood disorders, anxiety disorders, substance use disorders, impulsive control disorders, and eating disorders. It also included screening for psychosis and dementia, as well as the collection of demographic information, data on service use, and risk factors. Stage II focused the on the diagnoses of psychosis and dementia by trained psychiatrists using the Chinese version of SCID and the 10/66 Dementia Diagnosis Package. The data analyzed in this study were obtained from Stage I, using the CIDI. Data quality control procedures were implemented throughout the CMHS process, which consisted of computer-based logical checks, ordinal record checks, telephone checks by quality controllers and psychiatrists follow-up checks. Field procedures and data quality control are monitored and implemented in accordance with international standards (15–17).

### 2.3 Measurement

#### 2.3.1 Diagnose of mental disorders

The CIDI were used to obtain the diagnosis of mental disorders, including ADs, DDs, and alcohol use disorders (AUDs). Studies has shown that the Chinese version of CIDI is a reliable and validated, fully structured diagnostic interview designed to be conducted by trained lay interviewers to assess disease lifetime and 12-month disease state (15, 17). The ADs assessed included panic disorder, agoraphobia, special phobia, social phobia, obsessive compulsive disorder (OCD), generalized anxiety disorder (GAD). The DDs assessed included major depressive disorder(MDD), dysthymia, depressive disorder not otherwise specified.

#### 2.3.2 Sociodemographic factors

Sociodemographic information was obtained through the CIDI and including age (60-64, 65-69, aged 70 years and above), gender, growing areas (rural, urban), education level(illiterate/below primary school, Primary school, Junior high school and over), region (eastern, central, western), average annual household income (divided into three equal parts based on the actual sample: less than 10500¥,10500-42500 ¥, 42501¥ and over), and marital status was classified as married or cohabitating. Participants were asked about their height (in meter) and weight (in kilograms) to calculated Body Mass Index (BMI; less than 18.5, 18.5-23.9, 24 and above), in general, BMI= weight÷ height^2^.

#### 2.3.3 Non-communicable diseases

Data on NCDs were systematically collected using CIDI 3.0. Participants were queried about the presence of 16 types of NCDs experienced within the past 12 months. These conditions included arthritis or rheumatism, back and neck pain, headache, other chronic pain, seasonal allergies, stroke, heart attack, cardiopathy, hypertension, asthma, phthisis, chronic lung disease, diabetes, gastric or intestinal ulcers, epilepsy, and cancer. The total number of NCDs reported by each participant was calculated and categorized into three groups: one, two, or three or more chronic physical diseases.

### 2.4 Statistical analysis

The data was weighted using a comprehensive procedure. Each participant’s final analysis weight was calculated as the product of the sampling weight, non-response adjustment weight, post-stratification weight, and the trimmed weight adjustment. These weighting procedures follow well-documented methodologies, with detailed procedures and statistical analyses described in prior publications (15, 17). In this study, unweighted sociodemographic and weighted prevalence of anxiety disorders and depression are described. 95% Confidence Interval (CI) for prevalence estimates were calculated by the Taylor series linearization method, which adjusted for sampling weights and sample clustering. Univariate and multivariate logistic regression based on complex sampling was used to identify factors associated with 12-month ADs and DDs. For missing data, rigorous quality control minimized omissions, followed by thorough dataset cleaning. Median imputation was applied to questionnaire items with low missing rates. All analyses were conducted using SAS 9.4 and statistical significance level was set at P<0.05 (two-tailed).

## 3 Result

### 3.1 Characteristics of the sample

Among 5,428 older adults with NCDs, 52.1% were female, and 39.5% were aged 60 to 64 years. Approximately 78% of participants were married or living together with a partner. About 48.3% lived in urban areas, while 49.3% had no formal education or had education below primary school. Approximately 45% of participants had an average annual household income below ¥10,500, and 48.4% had a BMI ranging from 18.5 to 23.9. Approximately 38% of individuals suffered from three or more chronic physical diseases. Detailed sample characteristics are presented in **Table 1**.

**Table 1:** Sociodemographic characteristic and lifestyle of the sample (N=5428).

Variables	Frequency, n	Proportion
Gender		
Male	2602	47.9%
Female	2826	52.1%
Age (years)		
60-64	2143	39.5%
65-69	1479	27.2%
70 and over	1806	33.3%
Resident area		
Urban	2622	48.3%
Rural	2806	51.7%
Education		
Illiterate/below primary school	2675	49.3%
Primary school	1321	24.3%
Junior high school and over	1432	26.4%
Region		
Eastern	1857	34.2%
Central	1973	36.4%
Western	1598	29.4%
Household income		
<10500	2439	44.9%
10500-42500	1618	29.8%
>42500	1371	25.3%
Married/Cohabitating		
Yes	4258	78.4%
No	1170	21.6%
Alcohol use disorder		
Yes	210	3.9%
No	5218	96.1%
BMI		
<18.5	882	16.2%
18.5-23.9	2626	48.4%
>23.9	1920	35.4%
Number of NCDs		
1	1908	35.1%
2	1464	27.0%
≥3	2056	37.9%

BMI, Body Mass Index; NCD, Non-Communicable Diseases.

### 3.2 Prevalence of ADs and DDs

Among 5,428 older individuals with NCDs, 490 experienced ADs or DDs, resulting in weighted lifetime and 12-month prevalence of 15.0% and 9.5%, respectively. **Table 2** shows the weighted 12-month and lifetime prevalence of ADs and DDs among older adults with NCDs.

**Table 2:** Weighted 12-month and lifetime prevalence of ADs and DDs among adults aged 60 years and above with NCDs (N=5428).

Variables	12-month prevalence		Lifetime prevalence	
	Frequency, n	Weighted % (95% CI)	Frequency, n	Weighted % (95% CI)
Anxiety disorders	280	5.8 (4.6-7.0)	391	8.3 (6.4-10.1)
Panic disorder	33	0.8 (0.4-1.2)	45	1.1 (0.6-1.6)
Agoraphobia	17	0.2 (0.1-0.3)	25	0.6 (0.2-1.0)
Special phobia	147	3.1 (2.3-3.9)	203	4.2 (3.2-5.3)
Social phobia	26	0.6 (0.3-0.9)	37	0.9 (0.5-1.3)
Obsessive compulsive disorder	104	2.1 (1.4-2.9)	148	2.8 (2.0-3.6)
Generalised anxiety disorder	17	0.5 (0.2-0.9)	33	1.0 (0.4-1.5)
Depressive disorders	280	5.4 (4.3-6.5)	535	10.1 (8.2-12.0)
Major depressive disorder	228	4.4 (3.4-5.5)	376	7.2 (5.9-8.4)
Dysthymia	103	2.2 (1.5-2.9)	137	2.8 (2.1-3.6)
Depressive disorder not otherwise specified	46	0.8 (0.4-1.3)	144	2.7 (1.2-4.1)
Anxiety and depressive disorders	490	9.5 (7.8-11.1)	783	15.0 (12.4-17.5)

AD, Anxiety disorders; DD, Depressive disorders; NCD, Non-Communicable Diseases.

The lifetime prevalence of ADs was 8.3% and the 12-month prevalence of ADs was 5.8%. The two most prevalent ADs were specific phobia (SP; lifetime prevalence of 4.2% and 12-month prevalence of 3.1%) and obsessive-compulsive disorder (OCD; lifetime prevalence of 2.8% and 12-month prevalence of 2.1%). The lifetime prevalence of DDs was 10.1%, while the 12-month prevalence was 5.4%. The two most common DDs were major depressive disorder (MDD; lifetime prevalence of 7.2% and 12-month prevalence of 4.4%) and dysthymia (lifetime prevalence of 2.8% and 12-month prevalence of 2.2%).

### 3.3 Prevalence of ADs and DDs across different types of NCDs

ADs were most common in individuals with heart attacks (12%), chronic lung disease (11.2%), and other chronic pain (11%). The prevalence of ADs was 10% and 11% of individuals with headache and cardiopathy, respectively, and was below 10% in participants with gastric/intestinal ulcers, asthma, back and neck pain, seasonal allergies, stroke, phthisis, hypertension, diabetes, cancer, and epilepsy.

DDs are most commonly linked to heart attacks (13.8%), other chronic pain (13.2%), and cancer (13.1%). The prevalence of DDs among those with gastric/intestinal ulcers, epilepsy, chronic lung disease was between 10-11%, while it was less than 10% among individuals with cardiopathy, headache, asthma, back and neck discomfort, arthritis/rheumatism, diabetes, seasonal allergies, hypertension, stroke, and phthisis.

Older individuals with a history of heart attack (21%) or other chronic pain conditions (20%) had a higher prevalence of any ADs or DDs than those in other NCDs groups. **Table 3** shows weighted 12-month prevalence of ADs and DDs across different types of NCDs among adults aged 60 years and above.

**Table 3:** Weighted 12-month prevalence of ADs and DDs across different types of NCDs among adults aged 60 years and above.

NCDs		ADs		DDs		Anxiety or depressive disorders	
		n	Prevalence % (95%CI)	n	Prevalence % (95%CI)	n	Prevalence % (95%CI)
Arthritis/rheumatosis		144	6.5 (4.6-8.3)	153	7.3 (5.8-8.9)	256	11.1 (9.1-13.1)
Back and neck discomfort		182	7.3 (5.7-8.9)	191	8.3 (6.3-10.2)	323	13.2 (11.0-15.5)
Headache		93	10.6 (7.5-13.7)	102	9.2 (5.8-12.6)	165	16.4 (12.6-20.2)
Other chronic pain		66	11.0 (6.8-15.2)	88	13.2 (8.6-17.8)	129	20.0 (14.7-25.2)
Seasonal allergies		28	7.2 (2.5-11.9)	26	6.0 (2.2-9.7)	44	10.4 (4.9-15.9)
Stroke		20	6.9 (2.6-11.3)	25	4.2 (1.9-6.6)	38	10.2 (5.4-15.0)
Heart attack		53	12.0 (6.8-17.2)	64	13.8 (9.0-18.6)	98	21.0 (14.9-27.1)
Cardiopathy		83	10.0 (6.4-13.6)	84	9.9 (7.3-12.5)	141	16.3 (12.2-20.4)
Hypertension		139	5.9 (4.2-7.7)	133	5.7 (4.1-7.2)	244	10.2 (8.0-12.4)
Asthma		33	7.6 (3.8-11.5)	39	9.1 (4.8-13.4)	61	13.7 (8.6-18.7)
Phthisis		10	6.5 (0.7-12.3)	12	3.4 (0.7-6.1)	20	9.1 (2.6-15.5)
Chronic lung disease		23	11.2 (5.1-17.3)	23	10.2 (3.7-16.7)	38	16.8 (10.0-23.7)
Diabetes		43	5.8 (3.8-7.7)	48	6.7 (3.4-10.0)	80	11.6 (7.9-15.3)
Gastric/intestinal ulcers		46	8.0 (4.6-11.3)	57	10.8 (7.2-14.3)	84	15.7 (11.2-20.2)
Epilepsy		2	4.7 (0.0-13.0)	3	10.3 (0.0-28.0)	4	14.4 (0.0-33.7)
Cancer		4	5.3 (0.0-12.2)	7	13.1 (0.0-26.8)	10	15.0 (1.1-28.8)
Any NCDs		280	5.8 (4.6-7.0)	280	5.4 (4.3-6.5)	490	9.5 (7.8-11.1)
Number of NCDs	1	46	3.6 (1.7-5.4)	45	2.3 (0.9-3.6)	84	5.0 (2.8-7.1)
	2	60	4.3 (2.8-5.8)	39	2.8 (1.6-4.0)	87	6.0 (4.4-7.7)
	≥3	174	8.9 (6.8-10.9)	196	10.1 (7.8-12.4)	319	16.0 (13.3-18.7)

AD, Anxiety disorders; DD, Depressive disorders; NCD, Non-Communicable Diseases.

### 3.4 Correlates of ADs and DDs

Associations between factors and the 12-month ADs and DDs are shown in **Table 4**. The prevalence of ADs and DDs was associated with gender, age, education, marital status, alcohol use disorders, and the number of chronic physical diseases. No significant associations were observed with occupation, type of insurance, or other variables.

**Table 4:** Sociodemographic and clinical correlates for 12-month ADs and DDs among adults aged 60 years and above (N=5428).

Variables	ADs			DDs			Anxiety or depressive disorders		
	n	Crude OR (95%CI)	Adjusted OR (95%CI)	n	Crude OR (95%CI)	Adjusted OR (95%CI)	n	Crude OR (95%CI)	Adjusted OR (95%CI)
Sex									
Female	163	1	–	178	1	–	297	1	–
Male	123	0.78 (0.54-1.14)	**0.67 (0.47-0.97)**	102	**0.65 (0.44-0.97)**	**0.64 (0.45-0.89)**	193	**0.72 (0.57-0.92)**	**0.65 (0.49-0.86)**
Age (years)									
60-64	130	1	–	127	1	–	215	1	–
65-69	85	0.64 (0.41-1.01)	0.88 (0.66-1.18)	81	0.89 (0.61-1.30)	0.80 (0.60-1.08)	151	0.83 (0.61-1.12)	0.93 (0.74-1.18)
70 and over	71	**0.44 (0.27-0.72)**	**0.60 (0.44-0.82)**	72	0.75 (0.52-1.08)	**0.54 (0.40-0.74)**	124	**0.58 (0.44-0.76)**	**0.60 (0.47-0.77)**
Education									
Illiterate/below primary school	146	1	–	144	1	–	249	1	–
Primary school	79	0.95 (0.66-1.37)	1.04 (0.77-1.41)	82	1.33 (0.95-1.87)	**1.37 (1.02-1.83)**	139	1.25 (0.94-1.66)	1.22 (0.97-1.54)
Junior high school and over	61	0.98 (0.61-1.59)	0.77 (0.55-1.08)	54	0.97 (0.56-1.66)	0.92 (0.65-1.30)	102	0.98 (0.69-1.39)	0.85 (0.65-1.11)
Married/Cohabitating									
Yes	225	1	–	189	1	–	359	1	–
No	61	1.23 (0.80-1.88)	1.02 (0.75-1.38)	91	1.50 (0.96-2.35)	**1.81 (1.37-2.40)**	131	1.28 (0.93-1.76)	**1.38 (1.10-1.73)**
Alcohol use disorder									
No	259	1	–	264	1	–	455	1	–
Yes	27	**2.47 (1.29-4.71)**	**3.02 (1.91-4.78)**	16	1.47 (0.92-2.35)	**1.89 (1.07-3.34)**	35	**2.02 (1.29-3.19)**	**2.50 (1.66-3.77)**
Number of NCDs									
1	46	1	–	45	1	–	84	1	–
2	60	1.22 (0.64-2.32)	**1.72 (1.16-2.55)**	39	1.24 (0.58-2.66)	1.11 (0.72-1.73)	87	1.23 (0.75-2.01)	1.35 (0.99-1.85)
≥3	180	**2.62 (1.52-4.52)**	**3.72 (2.65-5.22)**	196	**4.87 (2.62-9.07)**	**4.16 (2.96-5.85)**	319	**3.64 (2.30-5.75)**	**3.86 (2.99-4.99)**

Bolded data indicate that these sociodemographic factors are statistically significant for prevalence; AD, Anxiety disorders; DD, Depressive disorders; NCD, Non-Communicable Diseases.

Older adults who were male (adjusted OR=0.67, 95% CI: 0.47-0.97) and those aged 70 years and above (adjusted OR=0.60, 95% CI: 0.44-0.82) were less likely to have 12-month ADs. In contrast, individuals with AUDs (adjusted OR=3.02, 95% CI: 1.91-4.78) and with two (adjusted OR=1.72, 95% CI: 1.16-2.55) or three or more (adjusted OR=3.72, 95% CI: 2.65-5.22) chronic diseases were more likely to suffer from 12-month ADs.

Similarly, being male (adjusted OR=0.64, 95% CI: 0.45-0.89) and aged 70 years and above (OR= 0.54, 95% CI: 0.40-0.74) were less likely to suffer from 12-month DDs. Those with primary education (adjusted OR= 1.37, 95% CI: 1.02-1.83), being not married or cohabitating (adjusted OR= 1.81, 95% CI: 1.37-2.40), with AUDs (adjusted OR= 1.89, 95% CI: 1.07-3.34) and with three or more NCDs (adjusted OR= 4.16, 95% CI: 2.96-5.85) were likely to suffer from 12-month DDs.

## 4 Discussion

To our knowledge, this is the largest nationwide study and the first research to investigate the prevalence and the correlates of ADs and DDs in older adults with NCDs in China.

### 4.1 The prevalence of ADs and DDs among community-dwelling older adults with NCDs

The lifetime and 12-month prevalence rates of ADs and DDs among community-dwelling older adults with NCDs are higher than those in the general population (16), suggesting that ADs and DDs are prevalent among older adults with NCDs in China. Researchers and clinical professionals should pay much attention to assess ADs and DDs when dealing with older adults with chronic diseases.

Consistent with findings in general population (16), our study demonstrates that ADs and DDs are the most common disorders among people with NCDs. For the ADs, SP and OCD were the two most common disorders. This may be explained by the fact that multiple physical diseases and the stress associated with physical illnesses are related to exacerbation of fearful responses to previous specific fearful scenarios and obsessive-compulsive symptoms in older adults (18, 19). For DDs, older people with NCDs are likely to have MDD and dysthymia. This is also consistent with previous findings (20, 21). Limitations on daily activities due to physical illness, discomfort from medications, disease recurrence and fear of death are associated with the onset of feelings of despair and helplessness in older patients, which in turn develop into major depression (22). Moreover, psychological distress and social isolation due to physical diseases are associated with the onset of dysthymia (23). However, a study of older patients in primary care in Wuhan found that mild depression is more common than dysthymia (13), which differs from our findings. This discrepancy may be due to differences in the study population, study methodology, and the subtypes of depression assessed.

### 4.2 Common types of chronic diseases comorbid with ADs and DDs

Our study revealed that among 16 common NCDs, older adults with heart attack, other chronic pain, chronic lung disease and cancer are most likely to suffer from ADs and DDs. Therefore, it is imperative for clinicians and primary care physicians to priorities the mental health status of older adults afflicted by such conditions when managing their healthcare in the community.

In our study, heart attack patients had the highest 12-month prevalence of ADs and DDs. This may be related to the high mortality rate of heart attack and the stress survivors experience when confronted with the disease, which may generate fear, apprehension, and negative attitudes and beliefs, thereby increasing the risk of anxiety and depression (24, 25). In addition, heart attack may lead to enhanced toxicity of central monoamine neurons and impaired monoamine transmitter function, which may be associated with an increased risk of developing depression (24, 26).

Patients with chronic pain are more likely to experience anxiety and depression, which is related to their bidirectional correlation and a shared pathogenesis (27, 28). Moreover, chronic pain is one of the somatization symptoms of anxiety and depression, but older adults with psychiatric disorders whose main complaint was somatic pain were often misdiagnosed with other somatic disorders, leading to delayed treatment and excessive waste of healthcare resources.

Numerous studies have shown that anxiety and depression are prevalent in people with chronic lung disease, which aligns with our findings (29, 30). A systematic review showed that the prevalence of clinical anxiety in hospitalized patients with chronic obstructive pulmonary disease (COPD) ranged from 10% to 55%, and from 13% to 46% in outpatients (31). A study in South Korea on chronic airway lung disease patients aged 45 and above showed that 51% of the patients had clinically significant depressive symptoms (32). The emergence of anxiety and depression in older adults may be strongly correlated with the discomfort, sleep disorders and limitation of physical activity resulting from chronic lung disease (33).

A number of studies have demonstrated that older adults with cancer often suffer from DDs (34, 35), which is consistent with our research results. Cancer is a highly heterogeneous disease, and its disease characteristics, treatment methods, patients’ lifestyles and social support, demographic factors, comorbid chronic diseases, impaired physical function, and dependence in daily life are all strongly associated with the onset and progression of depression (34, 36).

### 4.3 Associated factors of ADs and DDs in older adults with NCDS

In this survey, the prevalence of ADs and DDs was significantly associated with gender. Among older adults with NCDs, females exhibited a higher likelihood of experiencing these conditions, which aligns with previous researches (37, 38). The distinctiveness of women in genetics and neuroendocrine aspects (38), along with the pressures from traditional Chinese social and family roles (39), might render older women more susceptible to anxiety and depression than men.

Our study found that the risk of ADs and DDs was lower in the group of aged 70 years and above compared with the younger age group among older adults with NCDs. Although chronic diseases are generally believed to exacerbate anxiety and depressive symptoms in older adults, this trend does not appear to apply to individuals aged 70 years and above. A controlled study of long-term cancer survivors revealed a higher prevalence of anxiety and depression in patients under the age of 70 compared to gender- and age-matched general population samples, while no significant difference was observed among those aged 70 years and above (40). The possible cause for this phenomenon might be that the majority of people aged 70 years and above have a higher level of psychological acceptance of physical diseases than those under the age of 70, and psychological acceptance of illness is a key protective factor in the depression and anxiety network (41).

In the current study, participants with AUD have a higher risk of suffering from ADs and DDs than non-users, which is similar to the results of previous studies (42, 43). The inadequate management of alcohol abuse among older adults and long-term chronic drinking increase the risk of chronic NCDs, which are the reasons for the high prevalence. The impact of AUD on neurological function have been associated with increased susceptibility to depression (44), and recurrent alcohol abuse and withdrawal symptoms increase the risk of anxiety, depression, and psychotic symptoms (43).

Similar to previous research findings (44), our research has revealed a positive correlation between the number of chronic NCDs and an increased risk of ADs and DDs among older individuals. In the current study, the presence of two or more physical diseases elevates the risk of suffering from ADs, while the existence of three or more physical diseases enhances the risk of developing DDs. This suggests that with the increase in the number of NCDs, the psychological pressure of patients gradually intensifies, and patients are experiencing more and more negative emotions, ranging from initial worries and fears to depression, helplessness, and despair.

## 5 Limitations

Although this is the largest study for evaluating the prevalence of ADs and DDs among community-dwelling older adults with NCDs in China, several limitations should be noted. First, as the cross-sectional design of this study, it is impossible to determine the causal relationship underlying the reported correlations. To compensate for this limitation, prospective cohort studies are needed in future studies to verify the causal relationship of the factors involved. Second, the research participants were general community residents, excluding closed institutional communities such as nursing homes and inpatients, who may be at higher risk for ADs and DDs. Moreover, CIDI’s fixed algorithms may miss mild symptoms in older adults due to cognitive and language barriers, risking underdiagnosis of depressive disorders not otherwise specified. This highlights the need for tailored tools in future research. Finally, the study’s reliance on self-reported data for NCDs introduces potential limitations, including underestimation (especially asymptomatic disorders) and misclassification of NCDs. Despite employing standardized instruments, recall bias, social desirability bias, and disproportionate attention or neglect of NCDs among older adults could have compromised the accuracy of reported NCD diagnoses and subtype classifications. These inaccuracies may have subsequently impacted the observed prevalence of anxiety and depressive disorders within the study population.

## 6 Conclusion

This national study revealed a high prevalence of ADs and DDs among community-dwelling older adults with NCDs in China. Notably, ADs and DDs were prevalent among older adults suffering from heart attack, other chronic pain, chronic lung disease, or cancer. Several factors were identified as being associated with increased risk of anxiety and depression. Thus, it is increasingly significant to concurrently strengthen the management of chronic NCDs and mental health among older adults. Healthcare personnel should closely monitor the signs and symptoms of anxiety and depression in older adults, conduct early screening, identification, and diagnosis and treatment of common types of ADs and DDs. Special attention should be given to women, individuals with alcohol use disorders, those with two or more chronic NCDs, those with low educational attainment, and those who are divorced or widowed. Early detection and treatment of psychological problems such as ADs and DDs in older adults are of great significance for reducing disability and mortality rates and enhancing overall health of the older population.

## Data Availability

The original contributions presented in the study are included in the article/supplementary material. Further inquiries can be directed to the corresponding authors.
